# Can selenium‐enriched spirulina supplementation ameliorate sepsis outcomes in selenium‐deficient animals?

**DOI:** 10.14814/phy2.14933

**Published:** 2021-07-20

**Authors:** Thomas Castel, Michaël Theron, Karine Pichavant‐Rafini, Anthony Guernec, Aurélie Joublin‐Delavat, Bleuenn Gueguen, Karelle Leon

**Affiliations:** ^1^ Université de Brest EA 4324 ORPHY UFR Sciences et Techniques Brest France; ^2^ CNRS Univ Brest UMS 3113 Plouzané France; ^3^ UMR 6538 Laboratoire Géosciences Océan CNRS Univ Brest Plouzané France

**Keywords:** GPx, lactates, selenium, sepsis, Spirulina, supplementation

## Abstract

In intensive care units, sepsis is the first cause of death. In this pathology, inflammation and oxidative status play a crucial role in patient outcomes. Interestingly, 92% of septic patients exhibit low selenium plasma concentrations (a component of antioxidant enzymes). Moreover, *Spirulina platensis*, a blue‐green algae, demonstrated anti‐inflammatory effects. In this context, the main purpose of our study was to analyze the effect of a selenium‐enriched spirulina after a selenium deficiency on sepsis outcome in rats. Sixty‐four rats were fed 12 weeks with a selenium‐deficient food. After 8 weeks, rats were supplemented (via drinking water) for 4 weeks with sodium selenite (Se), spirulina (Spi), or selenium‐enriched spirulina (SeSp). Sepsis was then induced by cecal ligature and puncture, and survival duration was observed. The plasma selenium concentration was measured by ICPMS. Expression of GPx1 and GPx3 mRNA was measured by RT‐PCR. Blood parameters (lactates and HCO_3_^−^ concentrations, pH, PO_2_, and PCO_2_) were analyzed at 0, 1, and 2 h as well as inflammatory cytokines (IL‐6, TNF‐α, IL‐10). Sodium selenite and SeSP supplementations restored plasma selenium concentration prior to sepsis. The survival duration of SeSP septic rats was significantly lower than that of selenium‐supplemented ones. Gpx1 mRNA was increased after a selenium‐enriched spirulina supplementation while Gpx3 mRNA levels remained unchanged. Furthermore, sodium selenite prevented sepsis‐induced acidosis. Our results show that on a basis of a Se deficiency, selenium‐enriched spirulina supplementations significantly worsen sepsis outcome when compared to Se supplementation. Furthermore, Se supplementation but not selenium‐enriched spirulina supplementation decreased inflammation and restored acid–base equilibrium after a sepsis induction.

**New findings**

1 **What is the central question of this study?**

Sepsis, the first cause of death in intensive care units, is associated with systemic inflammation and oxidative stress and 92% of septic patients are selenium deficient. Spirulina and selenium have shown promising anti‐inflammatory and antioxidant effects. We have therefore hypothesized that a selenium‐enriched spirulina supplementation could provide beneficial effects on sepsis outcome in selenium‐deficient animals.

2 **What is the main finding and its importance?**

On a basis of a Se deficiency, selenium‐enriched spirulina supplementations significantly worsen sepsis outcome when compared to Se supplementation. Furthermore, Se supplementation but not Spirulina nor selenium‐enriched spirulina supplementations decreased inflammation and restored acid–base equilibrium after a sepsis induction.

## 1 INTRODUCTION

Sepsis is the first cause of mortality in intensive care units (Rudd et al., 2020), but despite numerous clinical trials and experimental studies, its pathogenesis is still unclear (Singer et al., 2016). Sepsis appears to be associated with systemic inflammation, cardiovascular system impairments, hemodynamic alterations, and acid–base imbalances (Bagshaw et al., 2009; Bateman et al., 2015; Boissier et al., 2017; Levraut et al., 1998; Razazi et al., 2019). In addition, the activation of immune cells leads to a release of pro‐inflammatory mediators (such as tumor necrosis factor‐α [TNF‐α] or interleukin‐6 [IL‐6]) that triggers an overproduction of reactive oxygen species (ROS) (Blaser et al., 2016; Mantzarlis et al., 2017). Moreover, septic patients display macrocirculatory failure resulting in tissue hypoxia (Hallisey & Greenwood, 2019), decrease in cellular oxygen consumption and lactate production contributing to develop an oxidative stress and inflammation (Gattinoni et al., 2019; Pan et al., 2019).

Selenium, a trace element essential to human health, is a component of selenoproteins involved in several functions such as fertility (Pieczyńska & Grajeta, 2015), thyroid or cardiovascular function (Benstoem et al., 2015; Köhrle, 2015). Moreover, selenoproteins like glutathione peroxidase and thioredoxin reductase are involved in oxidative stress regulation (Hosnedlova et al., 2017). Furthermore, it has been shown that, in intensive care units 92% of septic patients are selenium deficient (Sakr et al., 2007) and this selenium deficiency could play a role in sepsis development (Cirino Ruocco et al., 2018; Forceville et al., 1998; Mertens et al., 2015; Weber et al., 2008). In fact, a selenium deficiency is associated with a worsening of clinical outcome, excess mortality (Alhazzani et al., 2013; Angstwurm et al., 2007; Huang et al., 2013; Landucci et al., 2014) and an increase of nosocomial infections (Forceville et al., 2007). Selenium supplementation has therefore been largely studied. Recent studies demonstrate that if selenium administration does not reduce 28‐day mortality and duration of mechanical ventilation (Bloos et al., 2016; Kong et al., 2013; Li et al., 2019; Manzanares et al., 2016), it reduces the duration of vasopressor therapy (Chelkeba et al., 2015; Li et al., 2019). The beneficial effect of selenium supplementation in septic patients is still subject to debate and further investigations are needed to understand its involvement.

*Spirulina platensis* is a filamentous blue‐green algae with high protein, vitamin, phycocyanin, antioxidant, and polyunsaturated fatty acid contents (Habib, 2008; Lamela & Rocha, 2000; Leema et al., 2010; Pereira et al., 2019). Spirulina has been reported to have antioxidant effects in many models (Abdelkhalek et al., 2015; Bashandy et al., 2016; Gargouri et al., 2018; Nasirian et al., 2018). Furthermore, spirulina showed promising anti‐inflammatory properties, especially by reducing pro‐inflammatory cytokine release (Abdel‐Daim et al., 2018; Morsy et al., 2019; Pham et al., 2019; Zahran & Emam, 2018). Interestingly, spirulina can also be enriched in several essential elements such as selenium (Chen et al., 2006; Li et al., 2003).

In this context, we hypothesized that a selenium‐enriched spirulina supplementation could provide beneficial effects on sepsis outcome in selenium‐deficient animals. The effects of sodium selenite, *S*. *platensis*, and selenium‐enriched spirulina supplementations were studied in rats after 8 weeks of selenium‐deficient diet and 4 weeks of supplementation. Sepsis was induced by cecum ligature and puncture and the survival rates, acid–base equilibrium, blood pro‐inflammatory cytokines, and Gpx expression were evaluated.

## 2 MATERIAL AND METHODS

### 2.1 *Spirulina platensis* powder

The *Spirulina* strain used in this study was *S*. *platensis*. Production and conditioning (as dried powder) of spirulina and spirulina enriched with selenium was carried out by TAM company (Plougastel). The selenium concentration in selenium‐enriched *Spirulina* was 55 µg Se/g of dry weight of *S*.* platensis*.

### 2.2 Ethical approval

This study was performed in accordance with the European recommendations (2010/63/EU), approved by the regional ethical committee (CEFEA B29‐019–08) and the French Ministry (18325_2018123119211520). The authors understand the journal ethical principles and acknowledge that this study complies with this animal ethics checklist. Pain and suffering were minimized during the entire experiment.

### 2.3 Experimental protocol

#### 2.3.1 Animals

Sixty‐four 3‐week‐old female Wistar rats (Janvier, SAS‐Le Genest St Isle) with an average weight of 97.5 ± 1.6 g were included in this study. Rats were raised under a 12 h light–12 h dark cycle at 21℃. Food and water were given ad libitum. Rats were allowed to adapt to environmental conditions for 1 week before the experiments.

#### 2.3.2 Diet and supplementation

**FIGURE 1:**
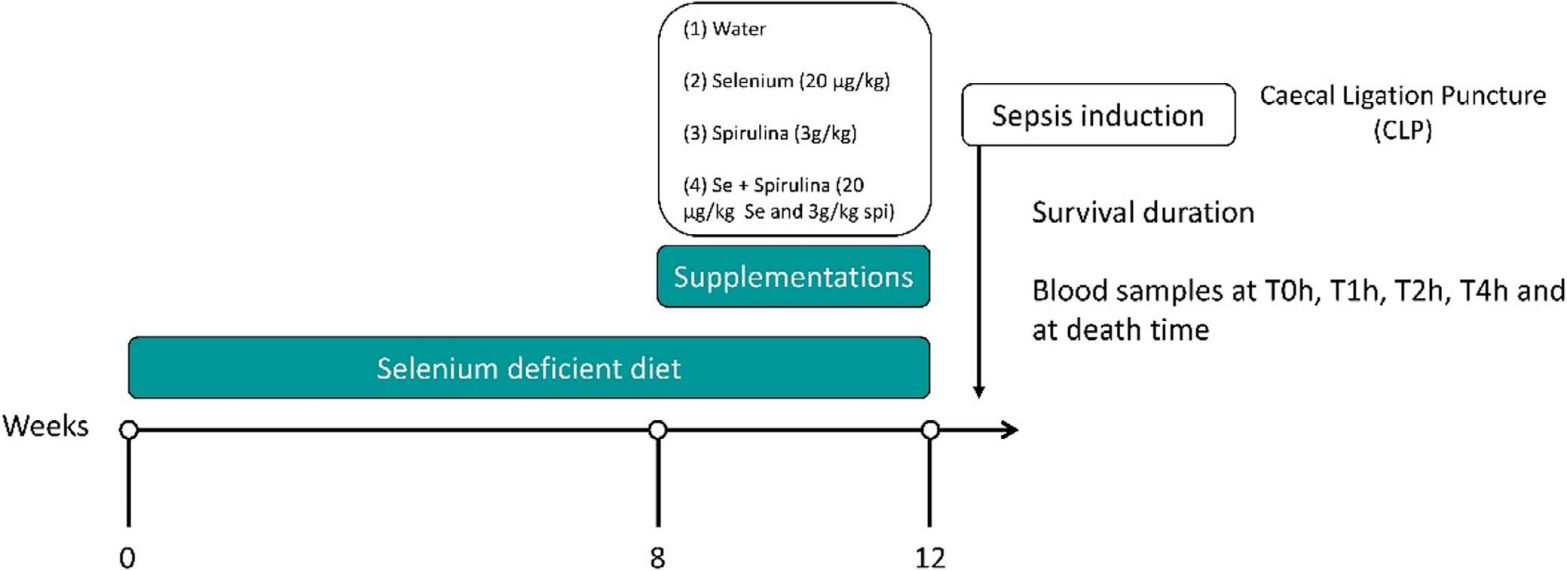
Experimental protocol

During the first 8 weeks of the experiment, animals were provided with tap water and food devoid of selenium (U8959P v.0170, SAFE, France) as can be seen in Figure 1. From week 8 to week 12, the rats (randomly assigned to one of the four following experimental groups—16 rats/group) were still fed with a selenium‐deficient diet but the beverage differed between the groups:

- D group (“D” for deficient) received tap water;
- Se group received selenium in water (Na_2_SeO_3_, 71950, Sigma Aldrich) at 20 µg Se/kg bw (body mass)/day;
- Spi group received *spirulina* in water at 3 g/kg bw/day;
- SeSP group received Se enriched *spirulina* in water at 3 g/kg bw/day, bringing 20 µg of selenium/kg bw/day.

All rats were weighted weekly, food, and drink intakes (given ad libitum) were measured twice a week during the 12 weeks of the experiment.

#### 2.3.3 Acute sepsis induction

Each group of 16 rats was randomly subdivided in two subgroups of 8 rats: sham and septic (described below).

In all cases, the rats were anesthetized by an intraperitoneal injection of a ketamine (100 mg/kg bw) and xylazine (10 mg/kg bw); a subcutaneous saline solution injection (3 ml/100 g bw) was performed and temperature was maintained at 38℃ (±0.5℃). The anesthesia was controlled all along the procedure and if needed, a half dose of ketamine xylazine was administered. A heparinized catheter (250 UI/ml) was inserted into the carotid to allow the collection of blood samples.

In the sepsis subgroups, cecal ligature and puncture (CLP) was performed as previously described (Léon et al., 2012). Briefly, after a laparotomy, the cecum was exposed and ligated in its middle. Next, the cecum was punctured once from side to side by a catheter (8 Gauge) and replaced in the abdominal cavity.

In the Sham subgroups, only laparotomy was done.

Survival times were evaluated for 6 h, after this delay, the animals (still under anesthesia) were euthanized by intracardiac puncture and blood draw.

### 2.4 Analytical procedure after acute sepsis induction

Arterial blood samples (300 µl) were collected via the carotid catheter immediately after the surgical procedure (T0h), then at T1h, T2h, and T4h (when the survival duration of rats made it possible). Seventy‐five microliters of blood was used for determination of selenoprotein expression by RT‐PCR, 75 µl was used extemporaneously for blood parameter measurements and 150 µl was used for plasma collection (by centrifugation at 2000 g for 5 min at 4℃) and subsequent cytokines quantification. Furthermore, at the animal death, total blood was collected for plasma isolation. Frozen samples were kept at −80℃ until assays.

### 2.5 Measured parameters after sepsis induction

#### 2.5.1 Measurement of plasma selenium concentrations

One hundred microliters of plasma, collected at the animal death, was weighed in PTFE vials, mixed with 2 ml of distilled 14N HNO_3_ and evaporated to dryness (on a hot plate overnight). One milliliter of 2.5% HNO_3_ was then weighted and added to the samples for ICP‐MS measurements. The concentrations of selenium were measured with a HR‐ICP‐MS Element XR (Thermofisher Scientific) at Pôle de Spectrométrie Océan (PSO) (IUEM/Ifremer) using indium as an internal standard for a drift signal correction. Selenium was measured at medium resolution to compensate for spectral interferences forming in the argon plasma. Calibration was performed with external standards; the detection limit was ~3 ng/g. Two procedural blanks were also processed following the above protocol and analyzed with the samples.

#### 2.5.2 Survival duration

Survival duration was determined in the four groups in septic and sham conditions. As mentioned earlier, 6 h after the beginning of the surgical procedure, surviving animals were euthanized.

#### 2.5.3 Selenoprotein expression

##### RNA isolation for RT‐PCR

Total RNA was isolated from total blood using the Nucleospin RNA Blood (740200.50, Macherey‐Nagel) according to a manufacturer's protocol adapted for total blood. Briefly, an enzymatic lysis was performed with 200 µl of Lysis Buffer and 5 µl of Proteinase K 15 min at room temperature. Then, 200 µl of 70% ethanol was added and the lysate was transferred into a Nucleospin column. After centrifugation at 11,000 g for 30 s, 350 µl of Membrane Desalting Buffer was added onto the column and centrifuged at 11,000 g for 30 s. The following RNA extraction steps (including DNAse treatment) were performed according to the kit procedure. At the end, RNA was eluted with 40 µl of DNAse/RNAse‐free water and stored at −80℃. RNA concentrations were measured with a SimpliNano^TM^ spectrophotometer (29‐0617‐12, GE Healthcare Life Sciences) and their purity was assessed using OD_260_/OD_280_ ratios. Their integrity was also checked by electrophoresis on a 1.5% agarose gel with ethidium bromide.

##### Quantification of Gene Expression by Real‐Time Reverse Transcriptase ‐PCR (RT‐PCR)

Blood mRNA levels of GPx3 and GPx1 were quantified by RT‐PCR. Total RNA (1000 ng) was reverse transcribed with the qScript cDNA synthesis kit (733‐1174, QUANTA BioSciences) containing a mix of oligo(dT) and random primers, dNTPs, Mg^2+^ and the reverse transcriptase. All cDNAs were then diluted 10‐fold for PCR experiments, which were realized with a 7500 Fast Real‐Time PCR (Applied Biosystems, Thermo Fisher Scientific). Target genes were amplified and quantified by SYBR^®^ green incorporation (EurobioGreen^®^ Mix qPCR 2x Lo‐Rox, Eurobio Ingen) with the primers given in Table 1.

The cycling conditions consisted of a denaturing step at 95℃ for 2 min, followed by 40–45 cycles of amplification (denaturation: 95℃ for 5 s; annealing/extension step: 60℃ for 30 s). Finally, a melting curve program was carried out from 60℃ to 95℃ with a heating rate of 0.1℃/s, showing a single product with a specific melting temperature for each gene and sample evaluated.

To obtain standard curves, all target genes were first amplified from a pool of RT products prepared with all rat samples. PCR products obtained were purified after electrophoretic separation on a 1.5% agarose gel using the Nucleospin gel and PCR Clean‐Up kit (Macherey‐Nagel). PCR products were then quantified using a SimpliNano^TM^ spectrophotometer before proceeding to a serial dilution from 10 pg/μl to 0.001 fg/μl. These seven‐point standard curves were used to determine the PCR efficiency of each primer pair.

The calculation of absolute mRNA level of a specific target gene was based on the PCR efficiency value (*E*) and the Threshold Cycle deviation (∆CT) of an unknown cDNA versus a control one (here, a pool of blood cDNA) according to the equation proposed by Pfaffl (2001): absolute mRNA level of a target gene = *E*_target_ *^∆^* ^CT(control−sample)^. For one gene, four runs were required to quantify the mRNA levels in all samples. Each run included no‐template controls and triplicates of the control cDNA. Inter‐assays variations were found to be <1.0%. To account for variations due to mRNA extraction and reverse‐transcription reaction, absolute mRNA levels obtained were corrected by 18s rRNA levels, used as a housekeeping gene. Thus, relative mRNA levels were expressed in arbitrary unit as ratios target genes/18S rRNA.

#### 2.5.4 Blood parameters measurement

Seventy‐five microliters of blood sample was immediately analyzed using a blood gas analyzer (ABL 80, Radiometer). Arterial blood pH, carbon dioxide partial pressure (PCO_2_), oxygen partial pressure (PO_2_), hematocrit, hemoglobin concentration, lactates, and bicarbonate concentrations were measured.

#### 2.5.5 Inflammation markers quantification

Plasma TNF‐α, IL‐10, and IL‐6 concentrations were measured by microElisa method using rat immunoassay kits (RTA00, R1000 and R6000B R&D Systems).

### 2.6 Statistics

All results are expressed as mean ± standard error of mean (SEM) or median ± interquartile range (IQR). Survival duration was analyzed using log‐rank test and illustrated in a Kaplan–Meier plot. Statistics were performed using GraphPad Prism v8.0.1 software. Normality was tested using the Shapiro–Wilk test. Adapted tests were then performed (ANOVA for repeated measures, Kruskal–Wallis test, and ANOVA 2 factors). ANOVA were followed by Tukey's post hoc test while Kruskal–Wallis test was followed by Dunn's post hoc test. A *p* value <0.05 was considered significant.

## 3 RESULTS

### 3.1 Effects of Se deficiency and supplementations (Se/Spi/SeSp) on rat growth

**FIGURE 2:**
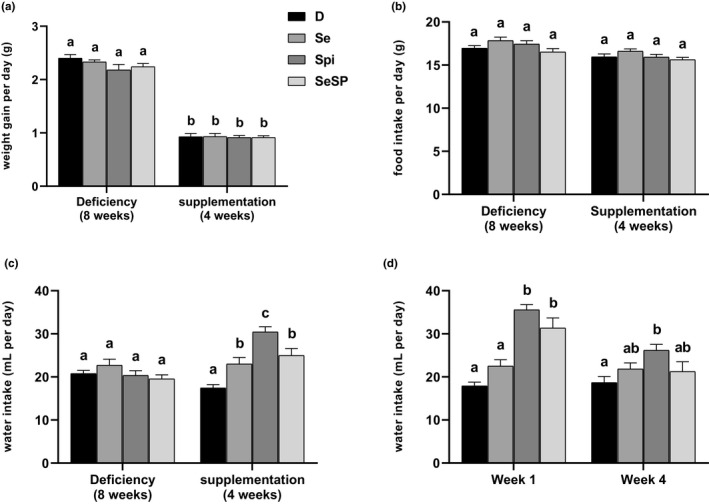
Effect of selenium‐deficient diet and sodium selenite, spirulina, or selenium‐enriched spirulina supplementations on rat growth. (a) Weight gain (g per day) in selenium‐deficient diet for 8 weeks and supplementations for 4 weeks. (b) Food intake (g per day) in selenium‐deficient diet for 8 weeks and supplementations for 4 weeks. (c) Water intake (ml per day) in selenium‐deficient diet for 8 weeks and supplementations for 4 weeks. (d) Comparison of water intake in week 1 and week 4 of supplementation. D, deficient, non‐supplemented group; Se, selenium‐supplemented group; Spi, spirulin‐supplemented group; SeSP, selenium‐enriched spirulina‐supplemented group. Data are expressed as mean ± SEM (*n* = 16 for each group). Different letters indicate significant difference between groups (*p *< 0.05)

The mean body weight gain during the experiment is presented in Figure 2a. During the 8 weeks of selenium deficiency, no significant difference between groups was observed. The supplementations did not affect the weight gain regardless of the supplementation type. As shown in Figure 2b, selenium deficiency and supplementations had no impact on the food consumption during the 12 weeks of experimentation.

However, water intake depended on the supplementation (see Figure 2c). During the 4 weeks of supplementation, rats supplemented with sodium selenite (Se) (23.00 ± 1.51 ml/day) and Se‐enriched *spirulina (*SeSP) (24.97 ± 1.60 ml/day) showed a significant increase in the amount of water consumed compared to the deficient (D) group (20.39 ± 0.75 ml/day). The group supplemented with *spirulina* (Spi) exhibited the highest daily water intake during this period (30.43 ± 1.21 ml/day). In order to determine whether the rise of supplementation consumption was linear during the 4 weeks of supplementation, a presentation of water intake at weeks 1 and 4 is shown in Figure 2d. It appears that Spi (35.59 ± 1.19 ml/day) and SeSP (31.36 ± 2.31 ml/day) triggered an increase in water consumption at week 1. This effect was reduced at week 4 and only Spi group maintains a significantly higher water intake compared to the D group (26.19 ± 1.33 vs 18.69 ± 1.39 ml/day).

### 3.2 Plasma Selenium concentration

**FIGURE 3:**
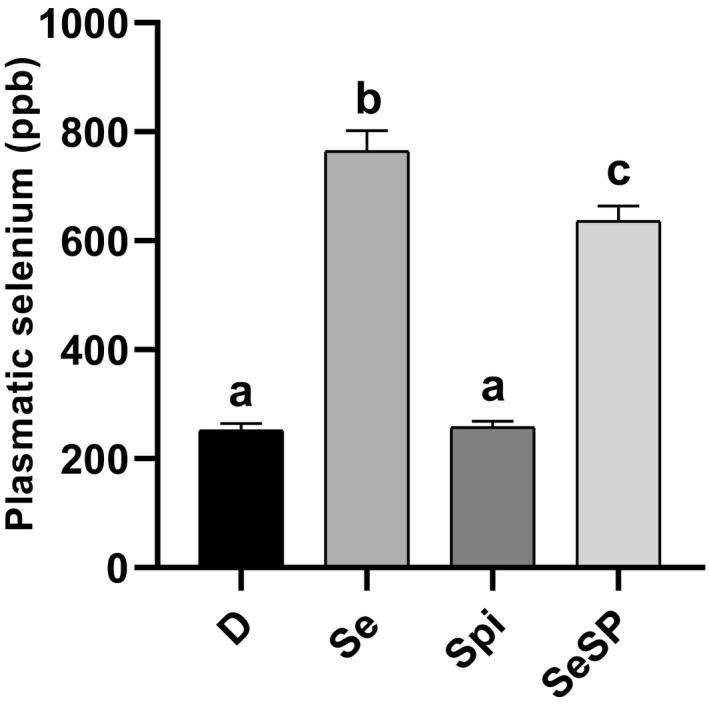
Plasma selenium concentration after 8 weeks of selenium‐deficient diet and 4 weeks of sodium selenite, spirulina, or selenium‐enriched supplementation. Data are expressed as mean ± SEM (*n *= 16 for each group). D, deficient, non‐supplemented group; Se, selenium‐supplemented group; Spi, spirulina‐supplemented group; SeSP, selenium‐enriched spirulina‐supplemented group. Different letters indicate significant difference between groups (*p *< 0.05)

Plasmatic Se concentration was measured from the blood sample performed at the death of the animals. The plasma selenium concentration in the D group was 252.27 ± 12.02 ppb (see Figure 3). Supplementation with spirulina (Spi group) did not induce any modification of this concentration. Se and SeSP supplementations led to a strong and significant increase in plasma selenium concentrations. Selenium concentration in Se group (764.74 ± 37.53 ppb) was significantly higher compared to SeSP group (636.33 ± 27.34 ppb).

### 3.3 Survival analysis

**FIGURE 4:**
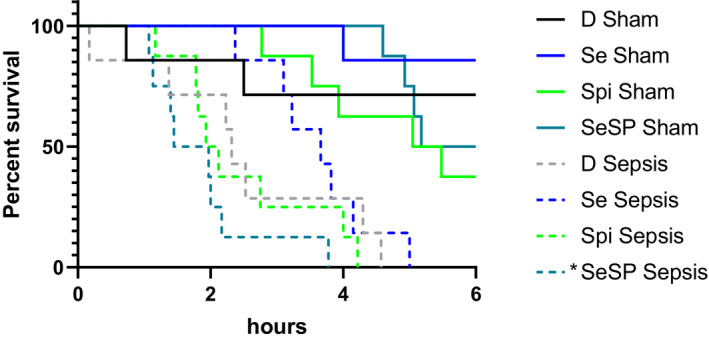
Survival duration rate after induction of sepsis or Sham operations. Data are expressed as percentage of T0 animal number (*n* = 8 for all group and condition except D sham *n *= 6 and D sepsis *n *= 7). D, deficient, non‐supplemented group; Se, selenium‐supplemented group; Spi, spirulina‐supplemented group; SeSP, selenium‐enriched spirulina‐supplemented group.*indicates a significant difference with Se Sepsis group (*p* < 0.05)

In Sham subgroups, the animals still alive at 6h were sacrificed and rat survival was not different from one group to the other (see Figure 4). However, at 6h the percentage of surviving rats ranged from 88% in the Se group to 50 and 40% in the SeSp and Spi groups, respectively; the difference between Se Sham and Spi Sham is at the limit of statistical significance (*p *= 0.06).

Sepsis induction significantly decreased the survival duration with every type of supplementation compared to Sham rats. Within the septic rat groups, a difference of survival duration was observed between the Se (3 h 37 min ±50 min) and SeSP groups (1 h 52 min ±21 min).

### 3.4 GPx1 and GPx3 mRNA levels

**FIGURE 5:**
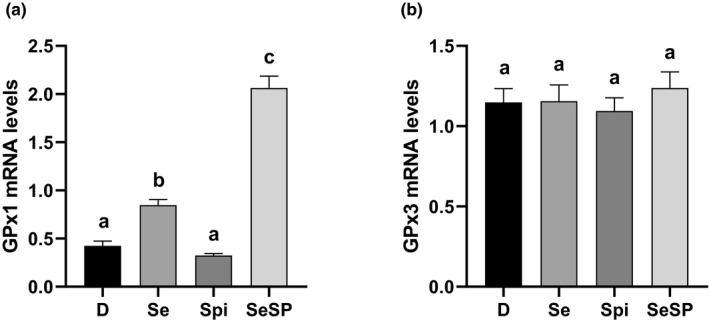
Plasma Gpx1 mRNA (a) and plasma Gpx3 mRNA (b) levels in D, Se, Spi, and SeSP rats. Data are expressed as mean ± SEM (*n *= 16 for each group). D, deficient, non‐supplemented group; Se, selenium‐supplemented group; Spi, spirulina‐supplemented group; SeSP, selenium‐enriched spirulina‐supplemented group. Different letters indicate significant difference between groups (*p *< 0.05)

From T0 to T4h, no significant difference throughout time appeared within the conditions for GPx1 and GPx3 mRNA levels; results were hence grouped (see Figure 5). There was no significant difference in GPx1 mRNA expression levels between the D and Spi groups (respectively, at 0.42 ± 0.05 and 0.32 ± 0.02). Se‐supplementation induced a slight increase of GPx1 mRNA (0.84 ± 0.06). In the SeSP group (2.06 ± 0.12), a fivefold increase appeared when compared to the D group. Concerning GPx3 mRNA levels, no significant difference was observed between conditions.

### 3.5 pH, P_CO2,_ plasma HCO_3_^−^, and lactate concentration

**FIGURE 6:**
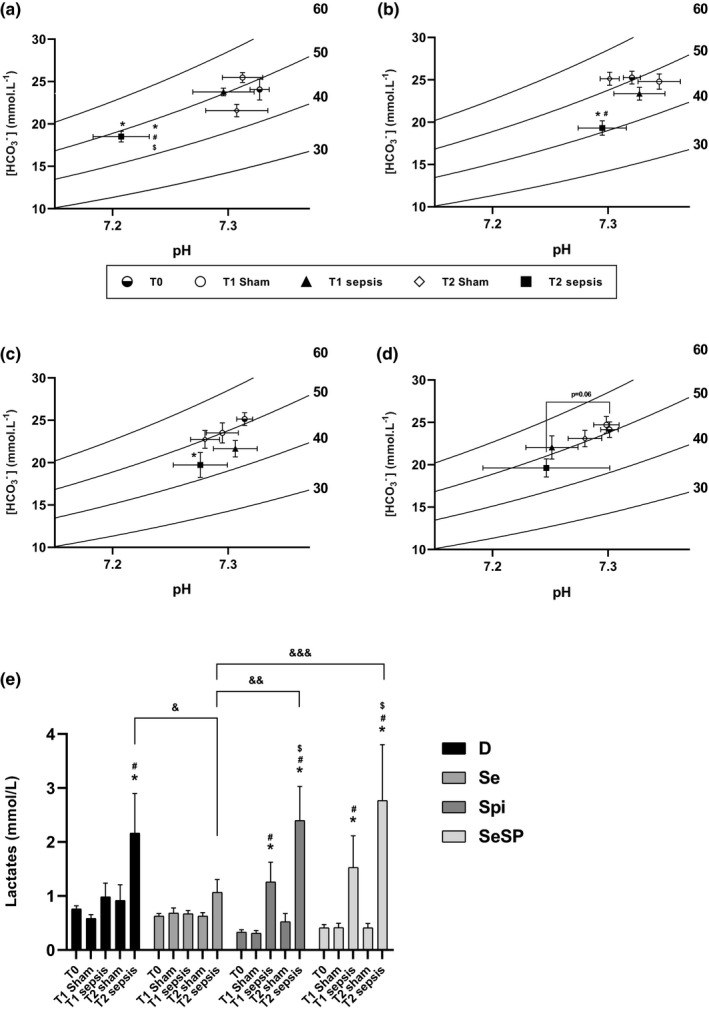
Davenport diagrams showing pH, PCO2, and bicarbonate (HCO3‐) concentrations of sham and septic rats in the D (a), Se (b), Spi (c), and SeSP (d) groups. Lactate concentrations in the D, Se, Spi, and SeSP groups at different time (e). Data are expressed as mean ± SEM. D, deficient, non‐supplemented group; Se, selenium‐supplemented group; Spi, spirulina‐supplemented group; SeSP, selenium‐enriched spirulina‐supplemented group. “*” indicates a significant difference with T0 in same group (*p* < 0.05). “#” indicates a significant difference with sham at the same time in the same group (*p* < 0.05). “$” indicates a significant difference with T1 septic in same group. “&” indicates a significant difference (*p* < 0.05)

Davenport diagrams, linking P_CO2_ with plasmatic HCO_3_^−^ concentration ([HCO_3_^−^]_p_) and pH, are shown in Figure 6. At T0, no significant difference was observed between groups for [HCO^3−^], P_O2_, P_CO2_, and pH (data not shown). Sham rats, at T1 and T2, displayed no significant difference with T0 in any condition. In the D group (Figure 6a), sepsis induced a significant acidosis at T2 (7.21 ± 0.02) compared to T0 (7.32 ± 0.01), T2 sham (7.30 ± 0.02), and T1 sepsis (7.30 ± 0.03). A decrease of [HCO_3_^−^]_p_ was also observed in D septic rats at T2 (18.5 ± 1.1 mmol/L) compared to T0 (23.83 ± 0.97 mmol/L). In the Se group (Figure 6b), sham rats displayed no significant difference of pH and [HCO_3_^−^]_p_ with T0. Furthermore, no significant pH difference was observed in septic rats while [HCO_3_^−^]_p_ was reduced in septic rats at T2 (19.3 ± 0.85 mmol/L) compared to T0 (25.25 ± 0.75 mmol/L). For the Spi groups (Figure 6c) pH was not different in sham and septic condition. At T2, in septic rats, [HCO_3_^−^]_p_ was lower (19.71 ± 1.47 mmol/L) than the T0 value (25.13 ± 0.76 mmol/L). Finally, in SeSP septic and sham rats (Figure 6d), no significant difference in pH and [HCO_3_^−^]_p_. was observed. Finally, pH in the Se septic group (7.32 ± 0.02) was higher compared to the SeSP septic group (7.25 ± 0.02) at T1h. At T2, pH was higher in the Se septic group (7.29 ± 0.02) when compared to the D septic group (7.21 ± 0.02).

No difference of plasmatic lactate concentrations was observed in the sham groups (whatever the time and the condition, see Figure 6e). In the D group, 2 h after sepsis induction, plasma lactate concentration (2.16 ± 0.73 mmol/L) was increased compared to T0 (0.76 ± 0.06 mmol/L), T1 sepsis (0.98 ± 0.25 mmol/L) and T2 sham (0.92 ± 0.28 mmol/L). In the Se group, no significant difference in lactate concentration with time in both sham and septic conditions was observed. Both the Spi and SeSP groups seem to exhibit a similar profile. In fact, lactate concentrations increased in septic groups at T1 (1.26 ± 0.36 and 1.52 ± 0.58 mmol/L, respectively) and T2 (2.4 ± 0.62 and 2.76 ± 1.03 mmol/L) compared to T0 (0.33 ± 0.04 and 0.41 ± 0.05 mmol/L, respectively), and T1 sham. Moreover, the Spi and SeSP groups showed a rise in lactate concentration compared to their own T1 sepsis and T2 sham. Finally, at T2h, the sepsis Se group showed lower lactate concentration (1.06 ± 0.23 mmol/L) than the T2 sepsis D (2.16 ± 0.73 mmol/L), Spi (2.4 ± 0.62 mmol/L) and SeSP groups (2.76 ± 1.03 mmol/L).

### 3.6 Hemoglobin, Hematocrit, and P_02_

No significant difference appeared for any of these parameters in any condition (data not shown). Mean hemoglobin concentration, hematocrit, and P_02_ were 13.4 ± 0.10 g/dL, 40.06 ± 0.31%, and 59.05 ± 0.84 mmHg, respectively.

### 3.7 Plasma IL‐6, TNF‐α, and IL‐10 concentrations

**FIGURE 7:**
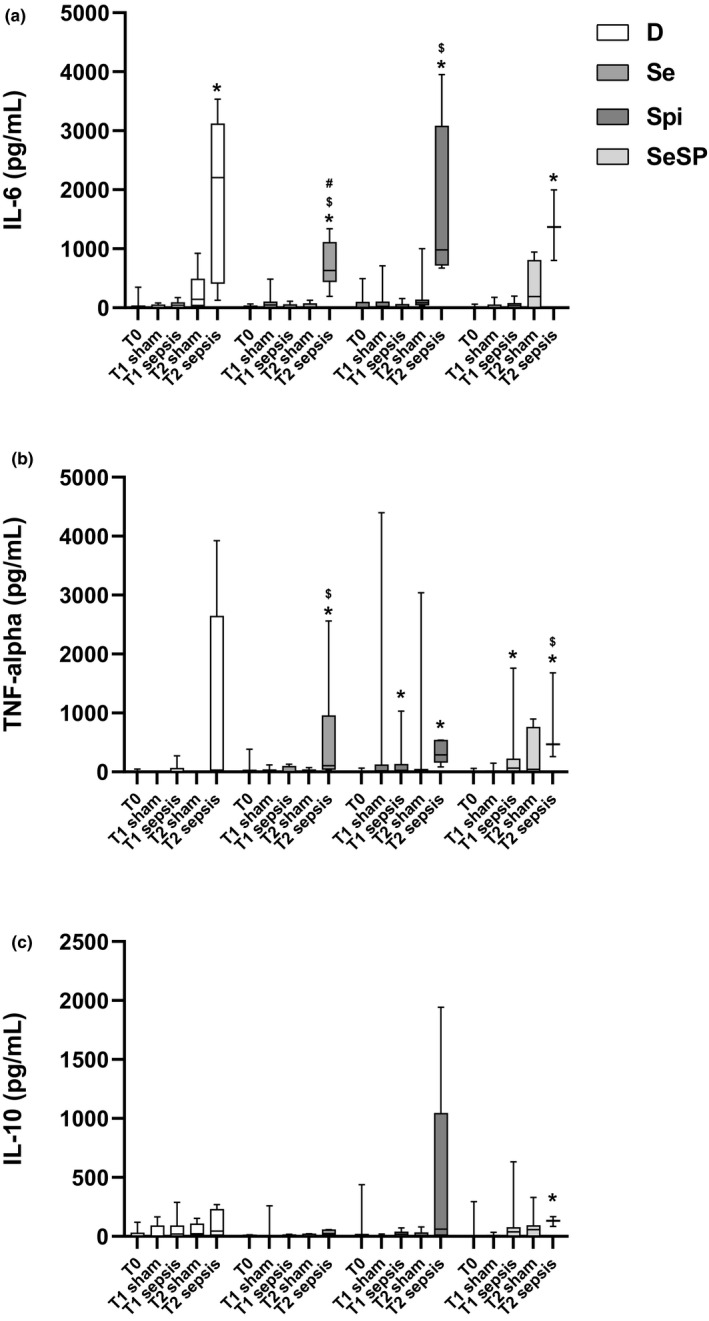
IL‐6 (pg/ml) plasma concentration (a), TNF‐α (pg/ml) plasma concentration (b), and IL‐10 (pg/ml) plasma concentration (c) in sham and septic rats at T0, T1h, and T2h. Data are expressed as median ± IQR (*n *= depending on survival rate). “*” indicates a significant difference with T0 in same group (*p* < 0.05). D, deficient, non‐supplemented group; Se, selenium‐supplemented group; Spi, spirulina‐supplemented group; SeSP, selenium‐enriched spirulina‐supplemented group; IQR, interquartile range. “#” indicates a significant difference with sham at same time in same group (*p* < 0.05). “$” indicates a significant difference with T1 septic in same group. “&” indicates a significant difference (*p* < 0.05)

No significant difference in plasma IL‐6 concentrations was observed between sham groups (Figure 7). In the D group sepsis, a rise was observed at T2 (2206.0 ± 2696.5 pg/ml) compared to T0 (0.0 ± 32.25 pg/ml). A similar difference (between T0 and T2 sepsis) was observed in all other groups.

Concerning plasma TNF‐α concentration, no significant difference between the sham groups was observed. In the D groups, no significant difference was observed between treatments. In the Se groups, plasma TNF‐α concentration was increased in T2 sepsis (106.2 ± 905.5 pg/ml) compared to T0 (0 ± 31.3 pg/ml) and T1 sepsis (0 ± 94.1 pg/ml). The same results were observed for Spi and SeSP rats.

No difference in plasmatic IL‐10 concentrations between the groups was observed, except for the SeSP septic group at 2 h that was significantly increased when compared to the corresponding T0.

### 3.8 DISCUSSION

Selenium deficiency is associated with an increase of mortality in intensive care units and spirulina is known to reduce inflammation in many models. Hence, the association of spirulina with a selenium supplementation as a selenium‐enriched spirulina, may combine antioxidant and anti‐inflammatory properties. In this context, the aim of our study was (on the basis of a Se deficiency) to investigate the effects of selenium‐enriched spirulina supplementation on sepsis outcomes.

In this study, weight gain and food intake were not affected by the supplementations. These results are consistent with those of Cases et al., (2001) that showed no difference in food intake after 6 weeks of selenium deficiency and 8 weeks of selenium supplementation. However, if spirulina beneficial effects on weight gain had previously been described (Khalil et al., 2018), no difference appears in our study. spirulina supplementation (Spi and SeSP groups), increases water intake during the first week of supplementation. As spirulina is cultivated in a saline environment, it could amplify the thirst sensation.

Plasmatic selenium concentrations were measured in order to validate our experimental procedures. According to Tanguy et al., (2012), in rat a plasma selenium concentration lower than 300 ppb corresponds to a selenium deficiency. This was the case in the non‐supplemented D group and in the Spi‐group. It can consequently be concluded that the protocol used has been efficient to induce a selenium deficiency. On this basis, a 4 weeks supplementation with sodium selenite (Se)‐ or selenium‐enriched spirulina made it possible to reach normal plasmatic selenium concentrations. Nevertheless, in SeSP‐supplemented rats, plasma selenium concentration was slightly lower than Se‐supplemented rats. Interestingly, several studies obtained similar results (Cases et al., 2001; Suzuki et al., 2006; Takahashi et al., 2017), while others did not evidence any difference between sodium selenite‐ and spirulina‐enriched supplementation (Falk et al., 2020; Zhang et al., 2020). When observed, the reduction of plasma selenium concentration in the SeSp group could be explained by the selenium form. In fact, during spirulina enrichment, 85% of selenite is present as organic selenium (Selenomethionine, SeMet) (Li et al., 2003) and SeMet when absorbed, has to be metabolized (either by trans‐sulfuration or conversion into methyselenol, Okuno et al., 2001) before its incorporation into selenoproteins.

The survival analysis after sepsis induction was performed with the sham and sepsis subgroups. Interestingly, in the sham groups, survival at 6h ranged from 88% in the Se group to 50% and 40% in the SeSp and Spi groups, respectively. The high survival in the non‐deficient sham rats (Se group) provide evidence for the validity of the surgical and anesthesia procedure but the survival seems to be strongly reduced when animals are supplemented with spirulina (*p *= 0.06). Similarly, the comparison of septic subgroups showed that Se rats displayed a better survival than SeSP rats. This point clearly need further investigation but if it is confirmed that spirulina reduces the positive outcome of surgical procedures and sepsis, it stresses a potentially strong negative effect of this nutraceutic product.

The effect of a selenium repletion after a selenium deficiency on the oxidative stress‐associated selenoproteins has been evaluated through plasma GPx1 and GPx3 mRNA levels. GPx1 but not GPx3 mRNA levels were increased in Se‐ and SeSP‐supplemented group compared to the groups without selenium supplementation (Spi and D groups). Moreover, the SeSP‐supplemented group shows a GPx1 mRNA content 2.5 times higher than the Se‐supplemented group. This results is consistent with those presented by Xia et al., (2005) who demonstrated that the highest human GPx activity was achieved with a 37 µg SeMet supplemental dose while the same levels of GPx activity required 66 µg of sodium selenite supplementation. Most of all, it highlights the selenoproteins expression hierarchy already described during selenium‐deficiency (Burk & Hill, 2015). In our model, GPx3 transcription seems to have priority on GPx1. However, our results also suggest that organic selenium absence downregulates GPx1 transcription (when compared to sodium selenite supplementation). Organic selenium could be better to enhance protein expression but does not appear to be sufficient to improve plasma selenium concentration and survival duration after sepsis induction compared to sodium selenite. It seems obvious that a better understanding of selenium form involvement in selenium repletion after a deficiency period is needed.

Acid–base disorders is a major issue in septic patients and in intensive care units, severe septic and septic shock patients exhibit elevated blood lactate concentration (Casserly et al., 2015; Levy, 2006). In fact, lactate concentration is considered as a good marker of sepsis severity and low lactate is associated with a reduction of mortality and a faster weaning of mechanical ventilation (Jansen et al., 2010; Nichol et al., 2011; Shapiro et al., 2005). In this experiment, a metabolic acidosis is observed 2 h after sepsis induction in the case of selenium deficiency. This metabolic acidosis was not observed in Se‐, Spi‐, and SeSp‐supplemented groups. In case of Se and Spi supplementation, the absence of acidosis can be explained by a respiratory compensation (since pH is maintained while pCO_2_ and/or [HCO_3_^−^] is decreased). Lactate concentration increased 1 h after sepsis induction in spirulina‐ and SeSP‐supplemented rats, but 2 h after sepsis induction in the D group. Spirulina appears consequently to be responsible for an early increase of lactate. This effect raises again the aforementioned concern of a potentially negative effect of spirulina in a surgical context. Interestingly, a sodium selenite supplementation totally prevents the increase in plasmatic lactate concentration, but the restoration of this disorder does not appear to be sufficient to significantly increase the survival of septic rats. During sepsis, hypovolemia leads to tissue hypoxia which in turn leads to the switch from aerobic to anaerobic metabolism. Thus, the pyruvate generated by glycolysis cannot be metabolized by mitochondria and is converted to lactate by lactate dehydrogenase (LDH). In our study, no changes in PO_2_, PCO_2_, and hemoglobin were observed over time (data not shown). Therefore, the sepsis‐associated hyperlactatemia occurring after 2 h of sepsis appears to have a metabolic origin and the hypothesis of a respiratory distress can be ruled out. Interestingly Se supplementation reduced lactate production but not SeSp despite their normal plasmatic selenium concentration: the selenium form appears to be important to face the metabolic disorders associated with sepsis. Since tissue hypoxia in septic patients appears to be insufficient to explain all the associated metabolic disturbances (Astiz et al., 1988; Hayes et al., 1994; Mouncey et al., 2015; Ronco et al., 1993), an evaluation of mitochondria activity, and of glycolytic enzyme involved in anaerobic ATP production could be pertinent to clarify the role of sodium selenite in lactate production.

In this study, pro‐inflammatory and anti‐inflammatory cytokines were measured. Indeed, as soon as a pathogen is detected by the immune system, pro‐inflammatory cytokines will be released (Faix, 2013), and, an early release of TNF‐α by macrophages (through TLR4 pathway) may induce disseminated intravascular coagulation, hypotension and multiple organ failure (Lv et al., 2014). IL‐6, also released by macrophages, is directly correlated with sepsis severity, inflammatory response and associated complications (Gouel‐Chéron et al., 2012). In our study, IL‐6 and TNF‐α expression displayed the same profile: 2 h after sepsis induction, a sharp increase was detected, with nevertheless one exception: for TNF‐α, in non‐supplemented rats where this increase was not significant (*p *= 0.058). These results are in accordance with clinical trials that demonstrate that the pro‐inflammatory cytokines release peak is reached 2 h after sepsis induction (Copeland et al., 2005). Nevertheless, beneficial effects of spirulina (Abdel‐Daim et al., 2015) and selenium (Duntas & Benvenga, 2015; Huang et al., 2013; Tan et al., 2013) supplementations had already been described but are not evidenced in our experiment. The control of the inflammation pathways is ensured by the release of anti‐inflammatory cytokines by the immune system and, in particular of IL‐10 (Chousterman et al., 2017). In our study, only SeSP rats reveal an increase of plasmatic IL‐10 level 2 h after sepsis induction. The others supplementations display extremely low IL‐10 level even 2 h after sepsis induction. SeSP supplementation could precociously activate the anti‐inflammatory pathway. Nevertheless, all together, these results do not suggest any clear beneficial effects of the different supplementations on inflammatory status during sepsis.

## 4 CONCLUSION

Sepsis is a complex pathology leading—via inflammation and oxidative stress—to potentially fatal multiple organ failures. Moreover, a selenium deficiency could play a role in sepsis evolution. In this context, and on a basis of a selenium deficiency, sodium selenite supplementation appears to improve metabolic disorders and acido‐basic equilibrium without reducing mortality. However, selenium‐enriched spirulina does not seem to be indicated in sepsis and raises questions about the selenium form involvement and its bioassimilation.

## CONFLICT OF INTEREST

The authors declare no conflict of interest.

## AUTHOR'S CONTRIBUTIONS

Castel T, Théron M, Pichavant‐Rafini K, and Léon K conceived and designed the experiments and contributed to the writing and revising of the article manuscript. Joublin‐Delavat A, Guernec A, and Gueguen B contributed to the acquisition, the analyses of the data and the revision of manuscript. All authors have seen and approved the final manuscript.

## DATA AVAILABILITY STATEMENT

The authors declare that data and material are available upon reasonable request.

5

**TABLE 1:** Primer sequences used for Real‐Time RT‐PCR analysis

Target gene	Abbreviation	Primer sequence (5’ to 3’)	Accession number	Data base
Glutathion Peroxidase 3	*Gpx3*	(F) CAAGAAGAACTTGGCCCATTC	BC062227	GenBank
		(R) GCTGGAAATTAGGCACAAAGC		
Glutathion Perixdase 1	*Gpx1*	(F) TGCAATCAGTTCGGACATCAG	NM_030826.4	GenBank
		(R) TTCACCTCGCACTTCTCAAAC		
Glyceraldehyde 3‐phosphate deshydrogenase	*Gapdh*	(F) GTATCCGTTGTGGATCTGACA	P04797	GenPept
		(R) CTGCTTCACCACCTTCTTGAT		
